# Transthyretin: A Transporter Protein Essential for Proliferation of Myoblast in the Myogenic Program

**DOI:** 10.3390/ijms18010115

**Published:** 2017-01-08

**Authors:** Eun Ju Lee, Smritee Pokharel, Arif Tasleem Jan, Soyeon Huh, Richelle Galope, Jeong Ho Lim, Dong-Mok Lee, Sung Wook Choi, Sang-Soep Nahm, Yong-Woon Kim, So-Young Park, Inho Choi

**Affiliations:** 1 Department of Medical Biotechnology, Yeungnam University, Gyeongsan 38541, Korea; gorapadoc0315@hanmail.net (E.J.L.); smriteep@gmail.com (S.P.); atasleem@gmail.com (A.T.J.); gjthdus0107@naver.com (S.H.); galoperichelle@gmail.com (R.G.); 2 Technology Convergence R&D Group, Korea Institute of Industrial Technology, Yeongcheon 770-200, Korea; lim2249@kitech.re.kr (J.H.L.); cowboyle@kitech.re.kr (D.-M.L.); 3 Department of New Drug Discovery and Development, Chungnam National University, Daejon 305-764, Korea; swchoi2010@cnu.ac.kr; 4 College of Veterinary Medicine, Konkuk University, Seoul 143-701, Korea; ssnahm@konkuk.ac.kr; 5 Department of Physiology, College of Medicine, Yeungnam University, Daegu 42415, Korea; ywkim@yumail.ac.kr (Y.-W.K.); sypark@med.yu.ac.kr (S.-Y.P.)

**Keywords:** myoblast, transthyretin, proliferation, differentiation

## Abstract

Irregularities in the cellular uptake of thyroid hormones significantly affect muscle development and regeneration. Herein, we report indispensable role of transthyretin (TTR) in maintaining cellular thyroxine level. TTR was found to enhance recruitment of muscle satellite cells to the site of injury, thereby regulating muscle regeneration. Fluorescence-activated cell sorting (FACS) and immunofluorescence analysis of TTR_wt_ (TTR wild type) and TTR_kd_ (TTR knock-down) cells revealed that TTR controlled cell cycle progression by affecting the expression of Cyclin A2. Deiodinase 2 (D2) mediated increases in triiodothyronine levels were found to regulate the expression of myogenic marker, myogenin (MYOG). Moreover, use of a coumarin derivative (CD) revealed a significant reduction in cellular thyroxine, thereby indicating that TTR play a role in the transport of thyroxine. Taken together, these findings suggest that TTR mediated transport of thyroxine represents a survival mechanism necessary for the myogenic program. The results of this study will be highly useful to the strategic development of novel therapeutics to combat muscular dystrophies.

## 1. Introduction

Skeletal muscle regeneration repairs muscle damage incurred during normal activities or by chronic disease or injury. This process involves multiple steps characterized by the activation of a primary myogenic population of stem cells, referred to as “satellite cells”, which leads to proliferation and differentiation followed by fusion with each other or with the existing myofibers to form multinucleated myotubes [1,2,3]. Satellite cells are normally quiescent, and express early myogenic transcription factors, such as paired box protein 7 (PAX7). These cells are activated to proliferate and generate committed progeny in response to a variety of stimuli, such as degenerative muscle diseases [3,4,5], and their activation requires the induction of myogenic differentiation (MYOD) and myogenic factor 5 (MYF5). The proliferation and differentiation of satellite cells are crucial to the maintenance of normal muscle mass during adult life and to the regeneration of new muscle fibers following injury [6]. Accordingly, disruptions of this process result in impaired skeletal muscle regeneration in response to injury and in long-term skeletal muscle wasting.

Thyroid hormones (THs) influence various tissues during development and post-natal life by modulating gene expressions [7,8,9]. These hormones are important regulators of gene expression and the impact of thyroid hormone signaling on skeletal muscle physiology has been well established. In fact, muscles are the major targets of THs, known to positively or negatively regulate the expressions of a broad range of genes at the transcriptional level [10,11]. Thyroid hormones are hydrophobic signaling molecules; therefore, their binding to distributor proteins such as thyroxine-binding globulin (TBG), transthyretin (TTR) and albumin facilitate their distributions to a large extent. Of the transporters present in blood, TBG exhibits the highest affinity for distribution of T4 and T3 (1.0 × 10^10^ and 4.6 × 10^8^ M^−1^, respectively), followed by TTR (7.0 × 10^7^ and 1.4 × 10^7^ M^−1^) and then albumin (7.0 × 10^5^ and 1.0 × 10^5^ M^−1^) [12]. Transit times for deliveries of THs to tissues are dependent on the binding affinities of TH distributor proteins, and thus, TTR (with intermediate affinity) compared with TBG and albumin is responsible for immediate delivery of THs to the tissues [8,12].

TTR is a protein that transports thyroxine and retinol binding protein to which retinol binds (highly conserved in terms of structure across a broad range of species) [12]. Although TTR synthesis is largely restricted to the liver, it has also been reported to be synthesized in the choroid plexus and in muscle tissues [8,13,14,15]. In mammals, TTR possesses higher binding affinity for T4 than for its active form T3, whereas in all other vertebrates TTR exhibits higher binding affinity for T3 than T4 [16]. On reaching its destination, T4 is acted upon by a family of deiodinases that either activate it via T4 to T3 conversion or inactivate it via conversion to T2 or rT3 [17]. T3 possess higher affinity than T4 for binding to receptors. After binding to thyroid receptors (TRs), T3 is translocated to nuclei, where it acts on the promoter regions of specific genes to induce dissociations of co-repressor molecules or association of co-activator proteins. In animal studies, TTR (TTR^−/−^) null mice displayed a delayed suckling-to-weaning transition, decreased muscle mass, delayed growth, and retarded longitudinal bone growth [18]. With high affinity for TRs, T3 was found to be associated with increasein the number and diameter of muscle fibers. Furthermore, T3 was found to play a role in the alteration of neonatal to adult myosin isoforms [18]. Mice with knock-down of both thyroid hormone receptor α and β (TRαβ^−/−^) showed significantly lower muscle weight at birth, which was the result of their having smaller and fewer muscle fibers [19,20]. Knowledge gained from animal models of altered thyroid hormone signaling revealed a more complex picture, in which thyroid hormones crucially regulate skeletal muscle homeostasis. Despite this, little is known about the factors involved in the modulation of TH availability at the cellular level or of the control of T3 level mediated by deiodinases in muscle and muscle progenitor cells. To investigate these issues, we studied the role of TTR in the transport of thyroid hormones during different stages of muscle development to clarify our understanding of the cellular pathways governing the progression of the muscle satellite cell lineage.

## 2. Results

### 2.1. Increases in TTR Expression and Hormone (T3 and Free T4) Concentration after Muscle Injury

**Figure 1:**
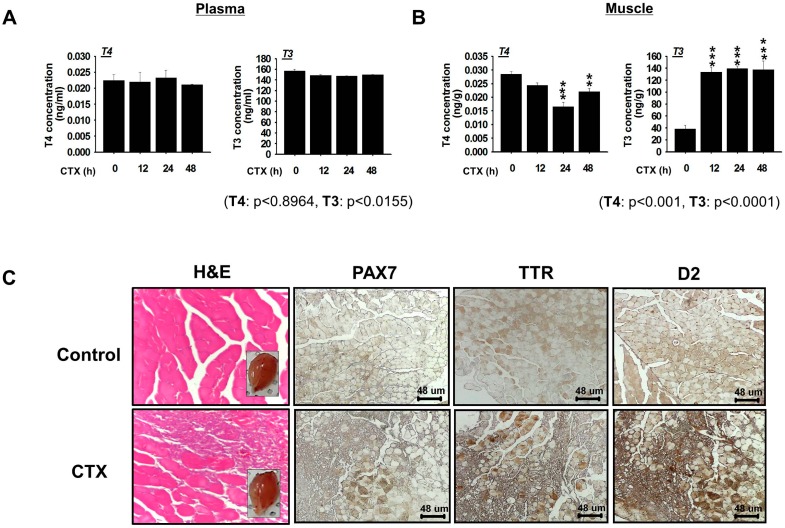
Relationships between muscle injury and thyroid hormone (T3 and free T4) concentrations. Tissue and plasma samples of mice subjected to cardiotoxin (CTX) injection were analyzed for the expressions of TTR and of the thyroid hormones, T3 and free T4. (**A**,**B**) Estimation of the concentrations of free T4 and T3 at early time points in plasma and muscle samples by ELISA. Concentrations of T3 and free T4 are measured as amounts (pg or ng) per gram of muscle tissue; (**C**) Transverse section of muscles after Hematoxylin–Eosin (H&E, 400×) and immunostaining with PAX7, TTR, or deiodinase 2 (D2) antibodies. The upper and bottom panel show non-injected (control) and CTX injected muscle, respectively. *p*-Values indicate statistical significance of the data (mean ± S.D., *n* = 3, **: *p* < 0.001, ***: *p* < 0.0001).

To establish the role played by TTR in the distribution of thyroid hormones during muscle regeneration, mouse gastrocnemius muscles were subjected to injury by cardiotoxin (CTX) injection. T3 and free T4 hormone plasma concentrations showed no significant change post-injection (Figure 1A). However, significant increases in T3 concentrations were observed in injured muscles at early (12, 24 and 48 h) time points (Figure 1B) compared with 0 h. Although the free T4 concentration changed to a lesser extent, significant changes with respect to T3 concentration were observed during the post injury recovery period. Loss of muscle tissue was observed at Day 3 post-injection. Furthermore, immunostaining with appropriate antibodies revealed increased protein expressions of PAX7, TTR and deiodinase 2 (D2) in the injured muscles (Figures 1C and S1).

### 2.2. Assessing the Role of TTR in T4 Delivery to Injured Muscle

**Figure 2:**
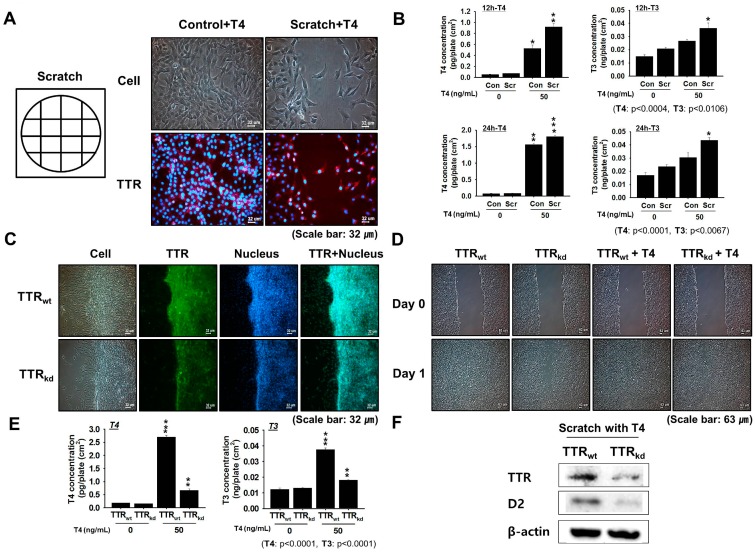
Effect of TTR knock-down on cell migration. Scratch testing was used to examine differences in cell migration. (**A**) TTR protein expression of control and scratched cells in the presence of T4 (red: TTR; blue: nucleus); (**B**) Difference in T3 and free T4 concentrations in normal and scratched samples at 12 or 24 h after T4 supplementation; (**C**) Analysis of scratched scrambled vector (control: TTR_wt_) and TTR shRNA transfected cells (TTR_kd_) conducted by staining with TTR antibody followed by 4′,6′-diamino-2-phenylindole (DAPI) nuclear staining (green: TTR; blue: nucleus); (**D**) Differences between the migration patterns of TTR_wt_ and TTR_kd_ cells in the presence or absence of T4; (**E**) Estimation of free T4 and T3 hormone levels in scratched and non-scratched cells in TTR_wt_ and TTR_kd_ cell cultures with or without T4 supplementation; (**F**) TTR and D2 protein expression was analyzed by Western blotting of TTR_wt_ and TTR_kd_ cells supplemented with T4. *p*-Values indicate statistical significance of the data (mean ± S.D., *n* = 3, *: *p* < 0.05, **: *p* < 0.001, ***: *p* < 0.0001).

Muscle regeneration involves the activation of satellite cells at the sites of injury and their subsequent proliferation and differentiation. To determine the role of TTR, which is known to act as a carrier of T4 in the recovery process, a time bound in vitro scratch study was performed. Cell proliferation experiments were performed to establish the role played by TTR in cell recovery when cell cultures were supplemented with T4 (50 ng/mL). Increased TTR expression along with T3 and free T4 hormone concentration was observed in scratched cells supplemented with T4 relative to non-scratched cells (Figure 2A,B). An in vitro scratch study of scrambled vector (control: TTR_wt_) and TTR shRNA transfected cell (TTR_kd_) cultures at early (12 h) time points revealed better recovery in TTR_wt_ cell cultures (Figure 2C). To confirm the roles of TTR and T4 during cell recovery, T4 was added to scratched TTR_wt_ and TTR_kd_ cell cultures. Cell migration rate were faster for TTR_wt_ than TTR_kd_ supplemented with T4 (Figures 2D, S2 and S3). Free T4 and T3 concentration in scratched and non-scratched cells was higher for TTR_wt_ than TTR_kd_ in cell cultures supplemented with T4. Furthermore, the expression of TTR and D2 decreased in response to the addition of T4 to scratched TTR_kd_ cells (Figure 2E,F). Together, these results confirm the importance of TTR as a T4 carrier protein during myoblast regeneration.

### 2.3. Induction in TTR Expression during Proliferation

**Figure 3:**
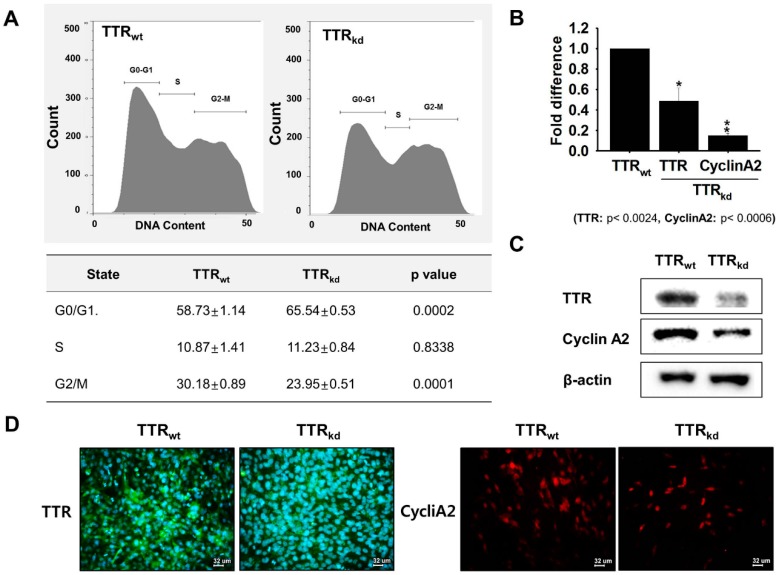
TTR expression during cell proliferation. TTR and CyclinA2 expressions during cell cycle progression of C2C12 cells: (**A**) difference in growth patterns of TTR_wt_ and TTR_kd_ cells as determined by FACS; (**B**,**C**) TTR and CyclinA2 expression in TTR_wt_ and TTR_kd_ cells by RT-PCR or Western blot; and (**D**) confirmation of expressional differences of TTR or CyclinA2 by immunocytochemistry (green: TTR; red: CyclinA2; Blue: Nucleus). *p*-Values indicate statistical significance (mean ± S.D., *n* = 3, *: *p* < 0.05, **: *p* < 0.001).

C2C12 cells undergo proliferation and differentiation to generate myotubes. Cell cycle analysis of TTR_wt_ and TTR_kd_ by FACS revealed significant differences between wild type and knock-down cells. The number of cells in G0/G1 phase for TTR_kd_ (65.54 ± 0.53) was higher than for TTR_wt_ (58.73 ± 1.14), while the number of cells in the G2/M phase for TTR_kd_ (23.95 ± 0.51) was lower than for TTR_wt_ (30.18 ± 0.89). However, no significant difference was observed between TTR_kd_ and TTR_wt_ in terms of the numbers of cells in the S phase (Figure 3A). To investigate TTR expression and its effects on proliferation, we analyzed CyclinA2 levels in TTR_kd_. TTR and CyclinA2 levels were lower in TTR_kd_ than in TTR_wt_ at the transcriptional and translational levels as determined by RT-PCR, Western blotting, and immunostaining (Figure 3B–D). Next, cells were scratched and incubated for 12 h with T4 supplemented media. TTR and CyclinA2 expression was up-regulated in scratched cells compared with non-scratched cells (Figure S4). These findings indicate that TTR mediated distribution of T4 regulates myoblast proliferation by affecting the expression of CyclinA2.

### 2.4. TTR Transport of T4 Enhanced Myoblast Differentiation

**Figure 4:**
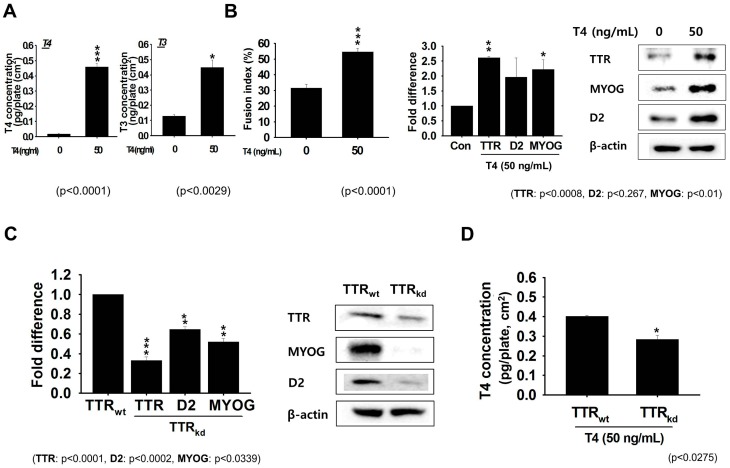
Estimation of T3 and free T4 concentrations with serum free media in C2C12 cells. C2C12 cells were incubated with serum free media for two days. (**A**) Difference in T3 and T4 concentrations in the presence or absence of T4 in serum free media; (**B**) Fusion indices determined on culture Day 2 with or without T4 supplementation. Expressions of TTR and MYOG as determined by RT-PCR and Western blot; (**C**) Differential expression of TTR, MYOG, and D2 in TTR_kd_ cells by RT-PCR and Western blot; (**D**) Analysis of T4 concentrations in TTR_kd_ and TTR_wt_ cells in the presence of T4. *p*-Values indicate statistical significance (mean ± S.D., *n* = 3, *: *p* < 0.05, **: *p* < 0.001, ***: *p* < 0.0001).

Thyroid hormones play important role in the cellular development, differentiation, and metabolism. We measured T3 and free T4 concentration in cells grown in serum-free media (Figure S5). Cells grown in serum-free media treated with T4 for two days showed higher concentrations of both hormones when compared to non-treated cells (Figure 4A). Furthermore, fusion indices and the mRNA and protein expression of MYOG, TTR, and D2 indicated that the addition of T4 enhanced cell fusion (Figure 4B). In addition, an analysis of TTR, D2, and MYOG mRNA and protein revealed lower expression in TTR_kd_ cells than TTR_wt_ cells (Figures 4C and S6). Interestingly, when the T4 concentration was measured in TTR_kd_ cells supplemented with T4, it was found to be lower than in TTR_wt_ (Figure 4D). TTR has high binding affinity for T4 and serves as its primary distributor protein in the muscle. The above findings indicate that TTR expression enhances myoblast differentiation by enhancing transport of T4 to the cell interior.

### 2.5. Competition for Binding to TTR during Myogenesis

**Figure 5:**
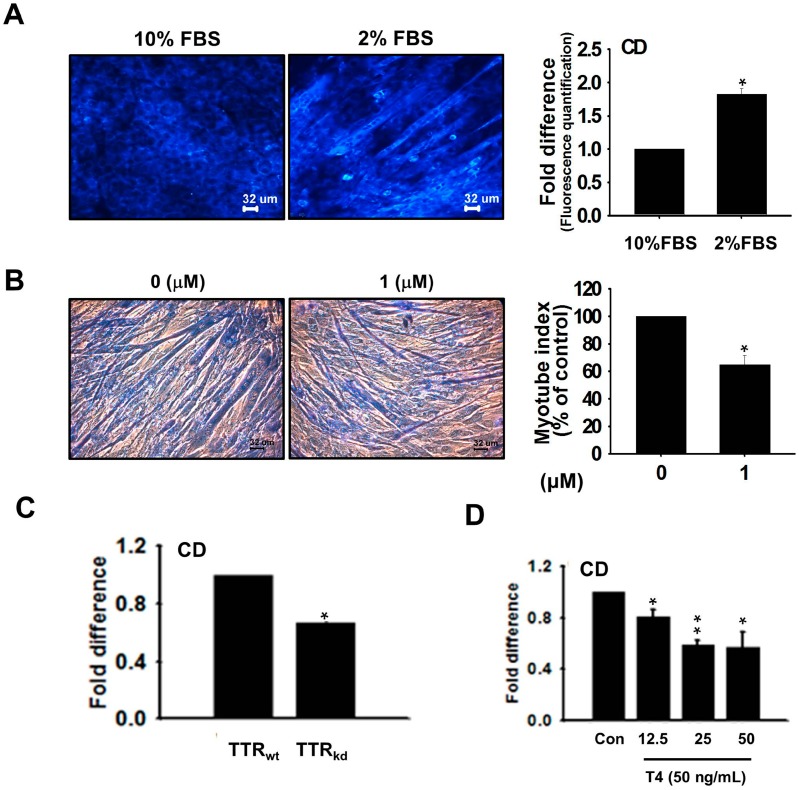
Effect of coumarin derivative (CD) on T4 to TTR binding. C2C12 cells grown in DMEM supplemented with 10% or 2% FBS were washed and incubated in serum-free media for 12 h. (**A**) Pictures showing fluorescence attributed to CD to TTR binding in C2C12 cells during growth in proliferation and differentiation media; (**B**) Giemsa staining showing myotube differences between C2C12 cells grown in differentiation media with or without CD (1 μM); (**C**) Graph showing CD intensity as fold differences in TTR_wt_ and TTR_kd_ cells; (**D**) CD intensity was measured in serum free media containing different concentrations of T4. *p*-Values indicate statistical significance (mean ± S.D., *n* = 3, *: *p* < 0.05, **: *p* < 0.001).

C2C12 cells cultured for proliferation (10% FBS) or differentiation (2% FBS) were treated with coumarin derivative (CD), a T4 competitive inhibitor known for the production of fluorescence on binding to TTR. Measurements of fluorescence intensity revealed higher values for differentiating cells than for proliferating cells (Figure 5A). However, the myotube index indicated that differentiating cells treated with CD showed less fused nuclear numbers than untreated cells (Figure 5B). TTR_kd_ cells treated with CD showed less myotube formation and fluorescence intensity, which was a result of less binding of CD to TTR_kd_ compared to TTR_wt_ cells (Figure 5C). A competition assay between T4 and CD for binding to TTR showed that increasing concentrations of T4 at a constant CD concentration reduced fluorescence intensity (Figure 5D).

## 3. Discussion

Technological advancements have led to the development of methods such as microarrays, and resulted in a shift towards global gene expression profiling [15,21]. When applied to muscle-related functions, information was obtained regarding the importance of genes with previously unknown functions, especially with regard to differential expressions in the myogenic program [8,9]. These findings led to increased interest in their roles during proliferation, differentiation and trans-differentiation. Studies pertaining to muscle development using different experimental models increase knowledge of the gene expressional changes during different stages of growth and development. The present study represents first step towards establishing their roles in muscle development.

Muscle damage occurs during normal activity and in response to chronic disease or injury. In this study, we found that TTR is associated with the delivery of THs required for skeletal muscle regeneration. With excess TTR (4.5 mM) in human plasma [22], concentrations of T3 and T4 remained unaltered in the plasma of mouse during early time points (12, 24 and 48 h) after injury. Normal TH levels are required for efficient muscle homeostasis, function, and regeneration [11,23], and this requirement for regeneration is fulfilled in part by thyroid gland secretion and by TH metabolizing enzymes (deiodinases) that regulate intracellular pools [24,25]. In a previous study, Larsen and Zavacki [26] reported that skeletal muscle largely relies on the endogenous conversion of T4 to T3 rather than on the serum T3. Similarly, our examination of T3 in mouse muscles showed increased T3 at 12, 24 and 48 h post injury. A significant change in mouse muscle TTR and D2 level indicates active conversion of TTR bound T4 to T3 by D2. Although less information is available on TTR maintenance of the T3 level in muscle, increases in the T3 level mediated by the D2 enzyme suggest its active role in the repair process. Furthermore, T4 to T3 conversion is associated with up-regulation of the activities of several transcription factors, such as MYOD, a well known regulator of muscle development and regeneration [27,28]. Together, these observations highlight the importance of TH delivery to muscle by TTR.

Skeletal muscle regeneration is an on-going process that is required to repair muscle damage incurred during normal activities and as a result of chronic disease or injury. To investigate this, we performed an in vitro scratch study using murine C2C12 cells to assess the provision of THs delivery by TTR to overcome muscle injury. In mouse C2C12 myoblasts, TTR expression was found to gradually increase during proliferation, and when cells were treated with T4, TH concentrations at 12 and 24 h post-treatment showed positive changes corresponding to T3 and T4 compared with the controls. shRNA knock-down of TTR was used to assess the effects of TTR on the regeneration process. Because an investigation of TTR null mice revealed unbound T4 in cerebrospinal fluid (CSF), suggesting that nothing can replace TTR in the transportation of T4 in CSF [29], a study of TTR knock-down was performed to assess its role in the transport of T4 in muscle. Knock-down of TTR in C2C12 cells was found to impair muscle regeneration, and scratch testing of C2C12 cells showed less recovery in TTR_kd_ cells than TTR_wt_ cells. Because TTR possesses higher binding affinity for T4 than T3, transit times for delivery of THs to tissues were analyzed using TTR_kd_ and TTR_wt_ cells. Supplementation with T4 was found to have a positive effect on TTR_wt_ cell migration as cells showed more rapid recovery than TTR_kd_ cells. These findings provide evidence of the importance of TTR for intracellular transport of T4 to support the maintenance process. We previously reported up-regulated expression of TTR during differentiation [15]. Moreover, a decrease in the intracellular T4 uptake accompanied by a decrease in the myotube number was observed in TTR_kd_ cells. Herein, active involvement of TTR in regeneration through regulation of the proliferation process was investigated. To clarify the involvement of TTR in skeletal myoblast proliferation, we used FACS to determine if cell proliferation was affected by TTR depletion. Under proliferative conditions, the proportion of cells in the G1 phase increased, but the proportion of cells in the S phase remained unaffected by TTR_kd_. In addition, cell proliferation (as assessed by G2/M transition) was lower in TTR_kd_ than in TTR_wt_ cells. These results indicate that TTR depletion somehow interferes with the proliferative potential of C2C12 cells. To obtain deeper insight into the mechanism operating at the proliferative stage, we analyzed cyclins in TTR_wt_ and TTR_kd_ backgrounds. The results revealed that knock-down of TTR had pronounced negative effect on the expression of Cyclin A2 at mRNA and protein levels. Furthermore, the expression corresponding to Cyclin A2 was found to be lower in TTR_kd_ than in TTR_wt_ cells. These findings provide clues regarding the regulatory mechanism by which TTR mediate T4 availability, thereby influence cell cycle progression.

Although the thyroid gland represents the first level of T3 availability to cells, the second level is constituted by TH transporters (MCT8, MCT10 and OATP1C1; organic anion transporting polypeptide 1c1) and TH metabolizing enzymes (deiodinases), which regulate intracellular T3 levels in a tissue-specific fashion [24,25]. To examine the effects of THs in more detail, we investigated the expression pattern of TTR in serum-free medium during differentiation of C2C12 cells. Upon supplementation of the medium with 50 ng/mL T4, we observed obvious increases in the intracellular levels of T3 and T4. THs are known to stimulate the expressions of several MRFs, including MYOG and MYOD [27,28]. To examine the transition from myoblasts to functional myotubes during differentiation, we analyzed the expression of differentiation marker (MYOG). After culturing C2C12 cells under differentiation condition for 2 days, analysis of the expression by RT-PCR and Western blotting showed increased expression corresponding to TTR and MYOG. For confirmation, we analyzed the expression patterns of TTR, D2 and MYOG in TTR_wt_ and TTR_kd_ backgrounds. In accordance with the above findings, the expression of these three genes was lower in TTR_kd_ than in TTR_wt_ cells. These findings showed that TTR increases MYOG levels, thereby promotes myotube formation. Further analysis of T4 in TTR_kd_ and TTR_wt_ cells revealed its levels were lower in TTR_kd_ cells. These results show that TTR participates in muscle development under both proliferation and differentiation conditions.

The observed increase in the TTR expression during proliferation, and its up-regulation during differentiation, indicates the importance of transporting T4 to maintain the cellular pool of T3 required for muscle development. TTR expression was assessed using CD (an analog of T4). Elevated fluorescence intensity was observed in CD undergoing complex formation with TTR in differentiating cells relative to proliferating cells, indicating the importance of T4 for the differentiation process. For further confirmation, we analyzed the myotube index in the presence of CD. The myotube index revealed that competition of CD with T4 for binding to TTR decreased myotube formation. The role of TTR, which functionally appears to be almost exclusively limited to the transport of T4 for its conversion to T3, appears to be well defined, and the contribution of TTR was checked using knock-down cells. These results show that knock-down of TTR decreased CD binding. Based on the increase in fluorescence observed in response to binding of CD to TTR, we conducted competition assays to assess its binding specificity to TTR. At constant CD concentration, increasing T4 concentration showed a decrease in fluorescence intensity, indicating that maintenance of the T3 intracellular pool requires T4 supply by TTR. These observations demonstrate the importance of TTR in the active transport of thyroid hormones required for muscle regeneration.

Together, these findings indicate that TTR plays an active role in the myogenic program by: (1) enhancing the recruitment of myoblasts to the site of injury; (2) assisting with the transport of thyroid hormones in muscle cells and in their subsequent conversion via the D2 enzymatic machinery; (3) controlling cell cycle progression through regulation of G0/G1-S transition mediated by Cyclin A2; and (4) contributing significantly to differentiation process by regulating the expression of MYOG. Based on these functions, TTR has great potential for use in the strategic development of novel therapeutics to combat muscular dystrophies.

## 4. Materials and Methods

### 4.1. Animal Experiments

C57BL/6 male mice (Koatech, Pyongtaec, Korea) were housed in a temperature-controlled room under a 12-h light cycle and fed standard chow diet with free access to water. A muscle injury model was produced as described by Kim et al. [30]. Briefly, cardiotoxin (CTX; 100 µL, 10 µM) was injected directly into the left gastrocnemius muscles under anesthesia (2.5% avertin: 10–15 µL/g body weight, i.p), while contralateral muscles were injected with phosphate buffered saline (PBS) and used as controls. Muscle samples from both legs were collected 12, 24, 48 h and 3 days post injection. The study was conducted in strict in accordance with the guidelines and protocols approved by the Institutional Animal Care and Use Committee of Yeungnam University College of Medicine (permit number: YUMC-AEC2015-006; 5 May 2015). After sacrifice, muscle tissues were collected from injured and control legs. CTX treated and control muscles were homogenized in sample dilution buffer (PBS with 1% BSA and 0.5% Tween 80) and homogenates were then centrifuged at 5000× *g* for 5 min at 4 °C. Supernatants were stored at −80 °C until required for total hormone estimation by ELISA. Organs were fixed by perfusion, collected and either stored at −80 °C until required for RNA and protein extraction or fixed overnight at 4 °C in paraffin-embedded tissue blocks for use in immunohistochemistry.

### 4.2. Cell Culture

Murine myoblast C2C12 cells (Korean Cell Line Bank, Seoul, Korea) were allowed to grow in DMEM (Dulbecco’s Modified Eagle’s Medium; HyClone Laboratories, South Logan, UT, USA) supplemented with 10% FBS (fetal bovine serum, HyClone Laboratories) and 1% PS (penicillin/streptomycin, Invitrogen, Carlsbad, CA, USA) at 37 °C in 5% CO_2_ atmosphere. At 70% confluence, media were exchanged with differentiation medium (DMEM + 2% FBS + 1% PS or DMEM + 1% PS), then incubated for 2 days. T4 (50 ng/mL) was added to the differentiation medium on Day 2.

### 4.3. TTR Gene Knock-Down

For knock-down, C2C12 cells grown to 30% confluence, were transfected with 1 ng of TTR shRNA (TTR_kd_) or a scrambled vector construct (control; TTR_wt_) using transfection reagent and transfection medium (Santa Cruz Biotechnology, Santa Cruz, CA, USA). Three days later, cells were selected by treating them with 2 μg/mL Puromyocin (Santa Cruz Biotechnology, Santa Cruz, CA, USA), after which they were allowed to grow to 70% confluence and then switched to differentiation media). Sequences of the shRNA constructs used are provided in Table S1. Knock-down efficiency was checked by analyzing mRNA and protein expression using RT-PCR and Western blot, respectively. The percentage difference in gene expression between TTR_wt_ and TTR_kd_ was used to depict the transfection efficiency of the shRNA knock-down construct.

### 4.4. Cell Cycle Analysis

Cell cycle analysis was performed by FACS. Briefly, TTR_wt_ and TTR_kd_ cells cultured in DMEM + 10% FBS + 1% PS for 3 days were trypsinized, collected, and centrifuged (2500 rpm, 5 min), after which the supernatants, containing trypsin, were discarded. Cells were then washed with ice-cold PBS and incubated overnight with 70% ethanol at −20 °C. The ethanol was subsequently removed by centrifugation (300 *g* for 5 min) and cells were washed with ice-cold PBS. Next, cells were treated with Muse cell cycle reagent (Merck Millipore, Darmstadt, Germany), incubated for 30 min in the dark at room temperature and then analyzed using an EasyCyte5HT unit (Merck Millipore).

### 4.5. In Vitro Injury Model

C2C12 cells were allowed to grow in DMEM + 10%FBS + 1% PS. Upon reaching 100% confluence, the middle portion of plates or six parts were scratched to remove cells. After scratching, media were switched to DMEM with 2% FBS and incubated for 12 or 24 h for further analysis.

### 4.6. Estimation of T3 and T4 Hormone Concentrations by ELISA

T3 (total T3) and T4 (free T4) concentrations of control or CTX injected muscle tissues and cell lysates (TTR_wt_ and TTR_kd_) were assayed using an ELISA kit (DRG International, Marburg, Germany). Briefly, homogenized muscle tissue or cell lysates with enzyme assay reagent were added to specific antibody-coated microtiter plates and then incubated for 30 min at room temperature. Enzyme conjugate was then added and plates were incubated for additional 30 min at room temperature. Mixtures were subsequently discarded after which plates were washed to remove unbound materials. Substrate solution was then applied and left for 20 min. The reaction was terminated by adding stop solution and color intensities were measured using a spectrophotometer at 450 nm (Tecan Group Ltd., Männedorf, Switzerland).

### 4.7. RNA Extraction and RT-PCR

Total RNA was extracted from cells using Trizol^®^ reagent (Invitrogen) according to the manufacturer’s protocol, dissolved in diethylpyrocarbonate-treated H_2_O, and then stored at −80 °C until required. cDNA was synthesized using a High capacity cDNA reverse transcription kit (Applied Biosystems, Foster City, CA, USA). Briefly, 2 µg of RNA in a reaction mixture volume of 20 µL was primed with random hexamer and reverse transcribed in a thermocycler programmed at 25 °C for 10 min, 37 °C for 120 min, and 85 °C for 5 min. Subsequently, 2 µL of cDNA product and 10 pmoles of each gene-specific primer were employed to perform PCR using a 7500 real-time PCR system (Applied Biosystems). Power SYBR^®^ Green PCR Master Mix (Applied Biosystems) was used as the fluorescence source. Primers were designed using the Primer 3 software (http://frodo.wi.mit.edu) using sequence information listed at the National Center for Biotechnology Information. Detailed information regarding the primer sequences used are provided in Table S2.

### 4.8. Immunocytochemistry

C2C12 cells cultured in covered glass-bottom dishes or tissue were stained using antibodies against TTR, CyclinA2 or PAX7. Briefly, cells were rinsed with PBS, fixed with 4% formaldehyde, and permeabilized with 0.2% Triton X-100 (Sigma Aldrich, St. Louis, MO, USA). Cells were then incubated with primary antibodies TTR (1:50; Santa Cruz Biotechnology) CyclinA2 (1:50; Abcam, Cambridge, MA, USA) or PAX7 (1:50; Santa Cruz Biotechnology) at 4 °C in a humid environment overnight. Secondary antibody (1:100; Alexa Fluor 488 or 594 goat anti-rabbit SFX kit; Molecular Probes, Invitrogen) was then applied for 1 h at room temperature, after which cells were rinsed with PBS. Nuclei were then counterstained with 4′,6′-diamino-2-phenylindole (DAPI; Sigma Aldrich) and pictures were taken using a fluorescent microscope equipped with a digital camera (Nikon, Melville, NY, USA).

### 4.9. Western Blot Analysis

Cells grown under different conditions were subjected to Western blot analysis. Briefly, cells were washed with ice-cold PBS and lysed in RIPA lysis buffer containing protease inhibitor cocktail (Thermo Scientific, Waltham, MA, USA). Total proteins were isolated by centrifuging lysates at 13,000 rpm for 10 min at 4 °C, and the protein concentrations were then determined using Bradford method. Separately, total proteins (40 μg) were heated at 90 °C for 3 min with β-mercaptoethanol (Sigma Aldrich), electrophoresed in 10% SDS-polyacrylamide gels, and then transferred to PVDF membranes (Merck Millipore), which were then blocked with 3% skim milk or BSA in TBST (Tris-Buffered Saline and Tween 20) for one hour. Membranes were subsequently incubated overnight with TTR (1:400), MYOG (1:400), CyclinA2 (1:400), D2 (1:400) or β-actin (1:1000) antibodies (Santa Cruz Biotechnology) diluted with 1% skim milk or BSA in TBS (Tris-Buffered Saline) at 4 °C. Blots were then washed in TBST, treated with horseradish peroxidase conjugated secondary antibody (goat anti rabbit or mouse antibody, Santa Cruz Biotechnology) for 1 h at room temperature, rewashed (as described above), and developed using Super Signal West Pico Chemiluminescent Substrate (Thermo Scientific).

### 4.10. Immunohistochemistry

The expression of TTR, PAX7 and D2 in mouse tissues was analyzed immunohistochemically. Briefly, paraffin-embedded tissue sections were deparaffinized, hydrated, quenched for endogenous peroxidase activity, blocked with 1% goat serum in PBS, and incubated with TTR (1:100), PAX7 (1:50) or D2 (1:50) antibody overnight at 4 °C. Samples were then incubated with HRP-conjugated secondary antibody (Santa Cruz Biotechnology). Positive signals were visualized by adding diaminobenzidine and hydrogen peroxide as substrates. A negative control experiment was also conducted by omitting the primary antibody. Following Hematoxylin–Eosin staining (H&E), stained sections of normal and injured muscles were examined for morphological changes using a light microscope.

### 4.11. Competition Assay Using a Coumarin Derivative (CD)

CD was synthesized using coumarin as a scaffold with a 3,5-dimethyl-4-hydroxyphenyl ring at its 3-position [31]. This ring is known to preferentially bind to the inner sub-site of the T4-binding pocket in TTR. Hence, CD was designed and synthesized to bind to T4-binding sites within TTR and to act as a sensor that emits fluorescence after binding correctly to folded tetrameric TTR in complex biological environments. The fluorescence spectrum of CD bound to TTR was recorded at 486 nm 10 min after CD supplement. Adequate fluorescence intensities were obtained using a CD concentration of 10 µM even after 3 h of treatment. C2C12 cells grown in 10% or 2% FBS for 4 days were washed and further incubated in serum-free DMEM for 12 h. TTR_wt_ and TTR_kd_ cells were allowed to differentiate in media containing 2% FBS for 4 days. For competition assays, cells were washed, incubated in serum free DMEM (to remove exogenous TTR and T4) for 12 h, then treated with CD (10 µM). In addition, cells were treated with T4 (12.5, 25 and 50 ng/mL) and incubated in the presence of increasing CD. After treatments, cells were washed with PBS and pictures were taken using a blue (420–495 nm) filter. Fluorescence intensities were quantified using the ImageJ software (National Institutes of Health, Bethesda, MD, USA).

### 4.12. Fusion Index

Fusion indices were calculated as described by Brigitle et al. [32]. Briefly, cell nuclei were stained with 0.04% Giemsa G250 (Sigma Aldrich) and pictures were captured randomly at three different locations. In addition, the number of nuclei in myotubes and the total number of nuclei in cells were counted in each field. The fusion index was defined as the number of nuclei incorporated into myotubes expressed as a percentage of the total number of nuclei.

### 4.13. Myotube Index

Cell nuclei were stained with 0.04% Giemsa G250 (Sigma Aldrich) and photographed at three different spots selected at random. The number of myotubes and total number of nuclei in each myotube were counted in each field. The five largest myotubes were selected from each picture, and the number of total nuclei in each myotube was calculated. Thus, the percentage nuclei incorporation per myotube was determined based on comparison of the control and treated groups.

### 4.14. Statistical Analysis

Normalized expression means were compared using Tukey’s Studentized Range (HSD) to identify significant differences in gene expression. Nominal *p*-values of less than 0.05 were considered statistically significant. RT-PCR data were analyzed by one-way ANOVA using PROC GLM in SAS ver. 9.0 (SAS Institute, Cary, NC, USA).

## Supplementary Materials

The following are available online at http://www.mdpi.com/1422-0067/18/1/115/s1, Table S1: shRNA information; Table S2: Primer information; Figure S1: PAX7 expression in normal or CTX injected muscles. PAX7 protein expression was observed with fluorescence in normal or CTX injected muscle (red: PAX7, blue: DAPI); Figure S2: Comparison of cell recovery in TTR_wt_ and TTR_kd_ cells. TTR_wt_ and TTR_kd_ cells were scratched, then incubated with DMEM + 2% FBS + 1% PS for 1 day. Cell recovery was measured in TTR_wt_ and TTR_kd_ cells; Figure S3: Effect of TTR knock-down on cell proliferation with T4; Figure S4: TTR, CyclinA2 and D2 expression with T4 in scratched cells. TTR, CyclinA2 and D2 expression was analyzed by RT-PCR in scratched or non-scratched cells with T4. *p*-Values indicate statistical significance of the data (mean ± S.D., *n* = 3); Figure S5: Differentiation in serum free media; Figure S6: Cell differentiation in serum free for TTR knock-down.
