# Supplementary material for: Transthyretin: A Transporter Protein Essential for Proliferation of Myoblast in the Myogenic Program
Source: Int J Mol Sci. 2017 Jan 8;18(1):115. doi: 10.3390/ijms18010115 (PMC5297749; doi:10.3390/ijms18010115)
Supplement: Supplementary file 1 [file ijms-18-00115-s001.pdf]

Supplementary Materials: Transthyretin: A Transporter Protein Essential for Proliferation of Myoblast in the Myogenic Program

Eun Ju Lee, Smritee Pokharel, Arif Tasleem Jan, Soyeon Huh, Richelle Galope, Jeong Ho Lim, Dong-Mok Lee, Sung Wook Choi, Sang-Soep Nahm, Yong-Woon Kim, So-Young Park and Inho Choi

Table S1. shRNA information.

**shRNA**		**Sequence**
**TTRshRNA**		**TTR shRNA Plasmid (m) Is a Pool of 3 Different shRNA Plasmids**
(SC-39716-SH)	sc-39716-SHA	GATCCCTGTAGACGTGGCTGTAAATTCAAGAGATTTACAGCCACGTCTACAGTTTTT
	sc-39716-SHB	GATCCGAAGATGCCGTGAAGCATTTTCAAGAGAAATGCTTCACGGCATCTTCTTTTT
	sc-39716-SHC	GATCCCACCTGCTATTTCATTCAATTCAAGAGATTGAATGAAATAGCAGGTGTTTTT

Table S2. Primer information.

Species	Gene	Accession No.	Product Size (bp)	Temperature (°C)	Sequence (F)	Sequence (R)
Mouse	*GAPDH*	NM_001289726.1	155	55	5′-tgctggtgctgagtatgtcg-3′	5′-caagcagttggtggtacagg-3′
	TTR	NM_013697.5	165	59	5′-tggacaccaaatcgtactgg-3′	5′-aattctgggggttgctgac-3′
	CyclinA	NM_009828.2	227	59	5′-ctgtctctttacccggagca-3′	5′-agtgatgtctggctgcctct-3′
	MYOG	M95800	185	59	5′-tccagtacattgagcgccta-3′	5′-caaatgatctcctgggttgg-3′
	D2	NM_010050	152	59	5′-gatgctcccaattccagtgt-3′	5′-caggtggctgaaccaaagtt-3′

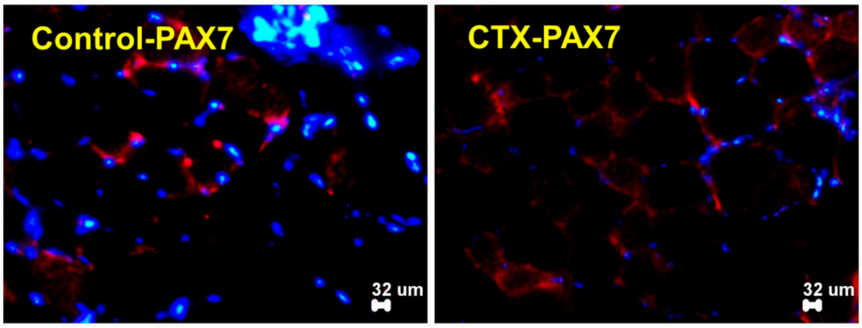

Figure S1. PAX7 expression in normal or CTX injected muscles. PAX7 protein expression was observed with fluorescence in normal or CTX injected muscle (Red: PAX7; Blue: DAPI).

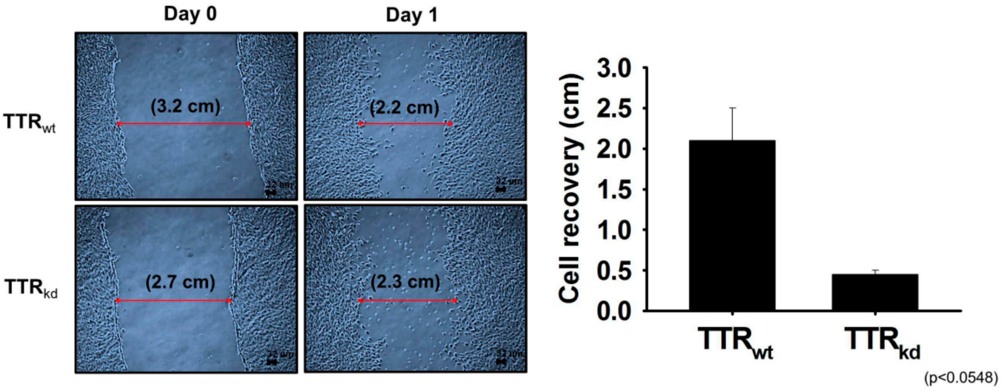

Figure S2. Comparison of cell recovery in TTR_{wt} and TTR_{kd} cells. Scratch was performed in TTR_{wt} and TTR_{kd} cells and incubated with DMEM + 2% FBS + 1% PS for 1 day. Cell recovery was measured in TTR_{wt} and TTR_{kd} cells.

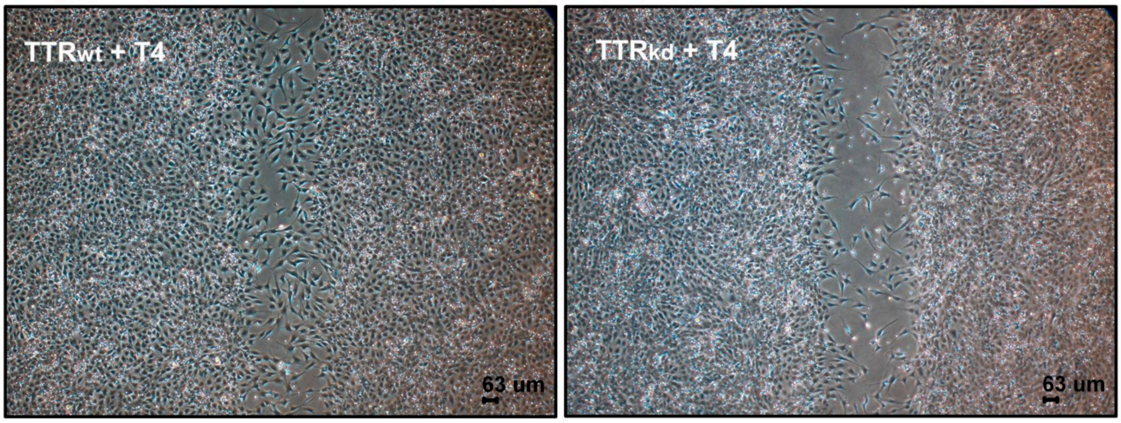

Figure S3. Effect of TTR knock-down on cell proliferation with T4.

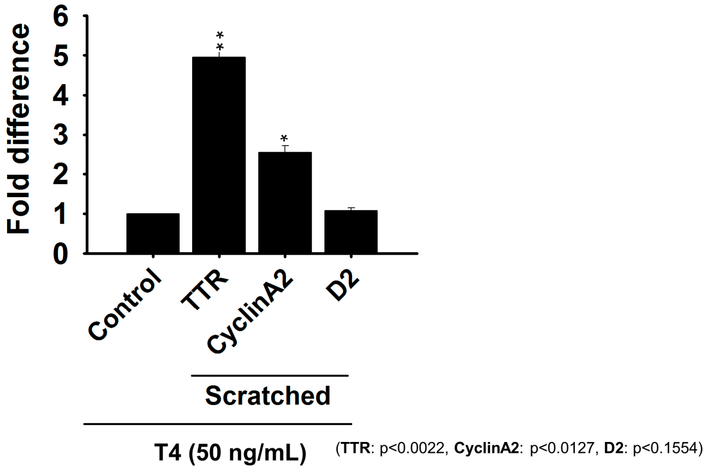

Figure S4. TTR, CyclinA2 and D2 expression with T4 in scratched cells. *TTR*, *CyclinA2* and *D2* expression was analyzed by real time RT-PCR in scratched or non-scratched cells with T4. *p*-values indicate statistical significance of the data (mean ± S.D., $n = 3$, *: $p < 0.05$, **: $p < 0.001$).

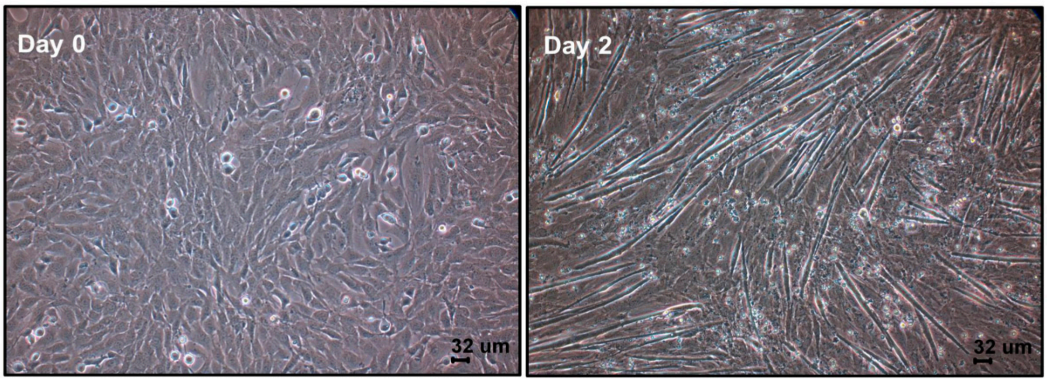

Figure S5. Differentiation in serum free media.

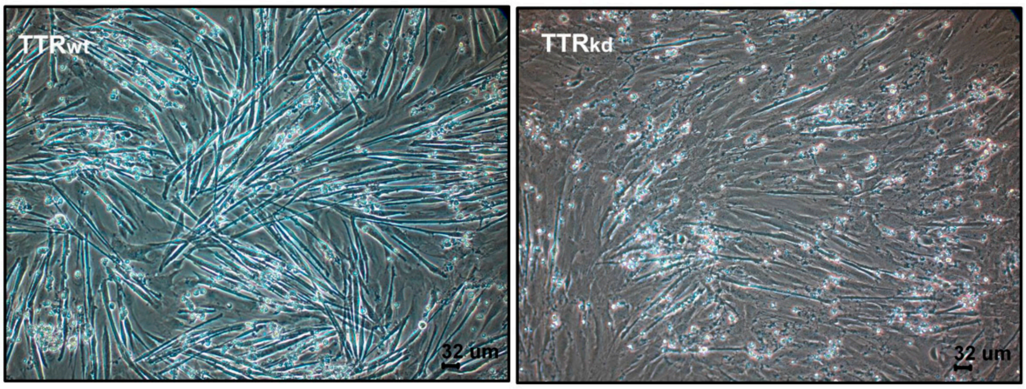

Figure S6. Cell differentiation in serum free for TTR knock-down.
